# Effect of JNK inhibitor SP600125 on hair cell regeneration in zebrafish (*Danio rerio*) larvae

**DOI:** 10.18632/oncotarget.10540

**Published:** 2016-07-12

**Authors:** Yingzi He, Chengfu Cai, Shaoyang Sun, Xu Wang, Wenyan Li, Huawei Li

**Affiliations:** ^1^ Department of Otorhinolaryngology, Key Laboratory of Hearing Science, Ministry of Health, EENT Hospital, Fudan University, Shanghai, China; ^2^ Department of Otolaryngology Head and Neck Surgery, the First Affiliated Hospital, Xiamen University, Xiamen, China; ^3^ Key Laboratory of Metabolism and Molecular Medicine, the Ministry of Education, Department of Biochemistry and Molecular Biology, Fudan University Shanghai Medical College, Shanghai, China; ^4^ Laboratory Center, Affiliated Eye and ENT Hospital of Fudan University, Shanghai, China; ^5^ Institute of Stem Cell and Regeneration Medicine, Institutions of Biomedical Science, Fudan University, Shanghai, China; ^6^ Key Laboratory of Hearing Science, Ministry of Health, EENT Hospital, Fudan University, Shanghai, China

**Keywords:** JNK, SP600125, hair cell regeneration, zebrafish, Wnt

## Abstract

The c-Jun amino-terminal kinase (JNK) proteins are a subgroup of the mitogen-activated protein kinase family. They play a complex role in cell proliferation, survival, and apoptosis. Here, we report a novel role of JNK signalling in hair cell regeneration. We eliminated hair cells of 5-day post-fertilization zebrafish larvae using neomycin followed by JNK inhibition with SP600125. JNK inhibition strongly decreased the number of regenerated hair cells in response to neomycin damage. These changes were associated with reduced proliferation. JNK inhibition also increased cleaved caspase-3 activity and induced apoptosis in regenerating neuromasts. Finally, JNK inhibition with SP600125 decreased the expression of genes related to Wnt. Over-activation of the Wnt signalling pathway partly rescued the hair cell regeneration defects induced by JNK inhibition. Together, our findings provide novel insights into the function of JNK and show that JNK inhibition blocks hair cell regeneration by controlling the Wnt signalling pathway.

## INTRODUCTION

Cochlear hair-cell loss in humans causes progressive and permanent hearing loss [1–3]. Nonmammalian vertebrates, however, are capable of regenerating lost sensory hair cells after injury [4–6]. The zebrafish (*Danio rerio*) has an important mechanosensory system – the lateral line system – that helps the fish avoid obstacles and predators [7]. The lateral line neuromasts are made up of a group of sensory hair cells and supporting cells [8], and the hair cells in neuromasts are similar to the mammalian inner ear sensory hair cells in terms of both morphology and function [9, 10]. Zebrafish are able to regenerate hair cells rapidly after damage, and robust regeneration of hair cells occurs within 48 hours [6, 11, 12]. The new hair cells in neuromasts usually arise through proliferation and differentiation of non-sensory supporting cells during the process of proliferative regeneration [6, 11–13]. Thus, numerous studies have used the lateral line system to investigate the mechanisms of hair cell differentiation and regeneration [14–17].

The c-Jun amino-terminal kinase (JNK) signalling pathway is a member of the large mitogen-activated protein kinase family. Upon activation, JNK executes its functions in cell growth, differentiation, proliferation, apoptosis, and inflammatory responses [18–21]. Subsequent studies suggested that JNK is implicated in the incidence and progression of cancer, the immune response in mammals, and apoptosis in a variety of experimental models. It has become an important and promising target for the treatment of a wide variety of diseases such as chronic inflammation, diabetes, and tumours [22–26]. The JNK family is encoded by three genes, *jnk1*, *jnk2*, and *jnk3*. The *jnk1* and *jnk2* genes are ubiquitously expressed, whereas the *jnk3* gene is restricted to the brain, heart, and testes [20, 27–29]. It has been reported that JNK signal is related to many physiological and pathological processes, such as neuron sprouting [30], tubulin dynamics in migrating neurons [31], and the progression of cancer [32]. More recently, JNK has emerged as an important regulator of the processes of regeneration. In planarians, the conserved JNK signalling cascade is required for regeneration of posterior tissues. Loss of JNK function blocks planarian posterior regeneration because the stem-cell dependent Wnt signalling expression fails to establish itself after posterior injury [33]. Two recent studies show that JNK activity is required for wound healing, for driving stem cell mitosis, and for correctly triggering cell death during planarian regeneration [34, 35]. However, the specific function of the JNK pathway in hair cell regeneration is still not well understood.

The purpose of this study was to investigate the effects of JNK on hair cell regeneration. We show that JNK inhibition with SP600125 reduced the numbers of hair cells, decreased cellular proliferation, and induced cell death in the zebrafish lateral line neuromast following neomycin-induced hair cell loss. We further provide evidence that SP600125 attenuated the expression of genes related to Wnt activation. The phenotype of regenerating hair cells induced by JNK inhibition can be partly rescued by over-activation of the Wnt signalling pathway. These results suggest that JNK supports the regenerative proliferation of hair cells by controlling the Wnt signalling pathway.

## RESULTS

### JNK inhibition disrupts the regeneration of lateral line hair cells

**Figure 1:**
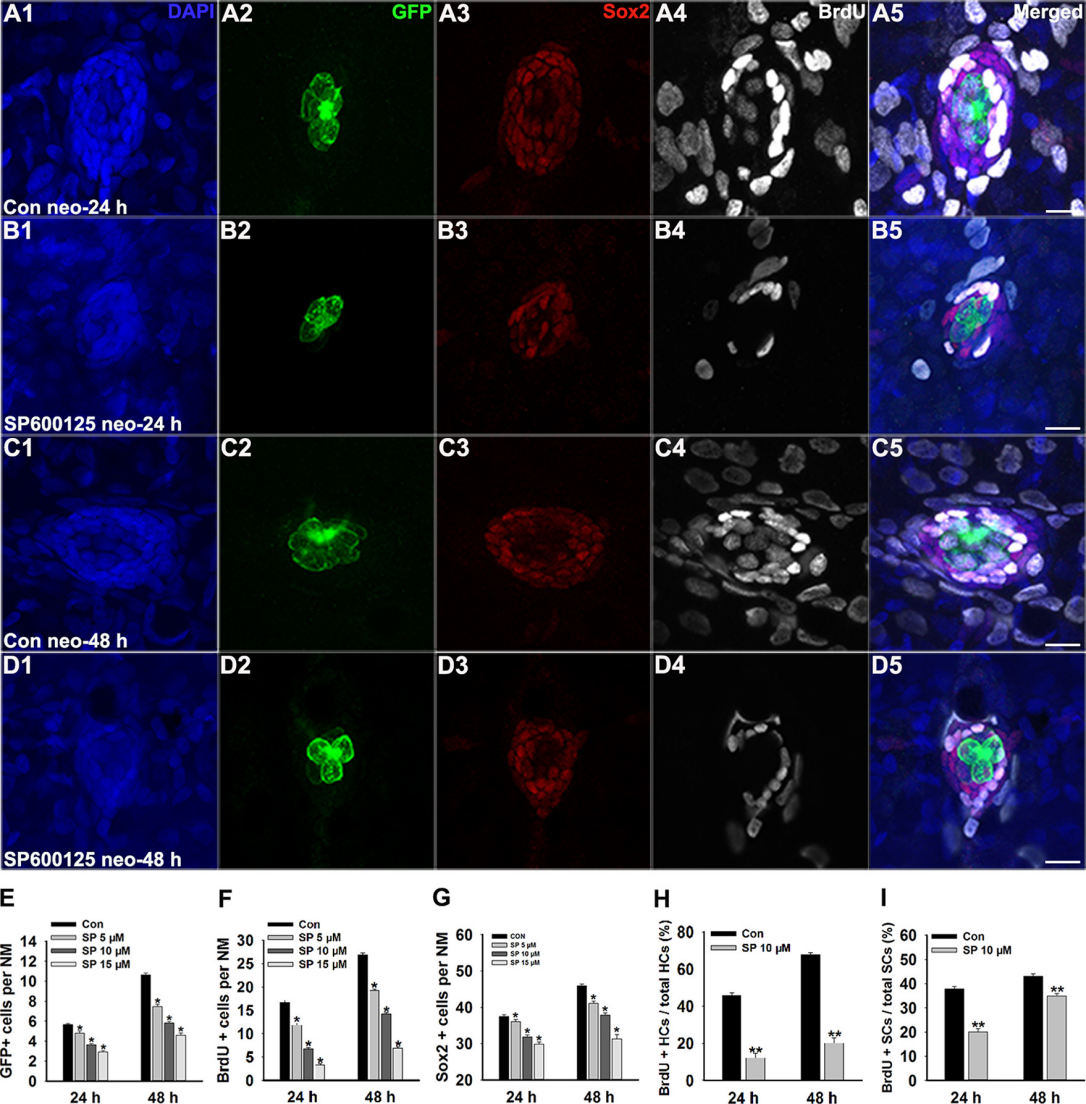
SP600125 decreases regeneration of hair cells in zebrafish lateral line neuromasts (**A**–**D**) We treated 5 dpf Tg(Brn3c:mGFP) zebrafish with 400 μM neomycin for 1 h and then treated them for 24 h or 48 h with 10 μM SP600125 and subsequently imaged GFP-positive hair cells (green), Sox2-positive supporting cells (red), and BrdU-positive replicating cells (white). SP600125 significantly decreased the numbers of GFP-positive hair cells and Sox2-positive supporting cells in neuromasts as well as reduced the proportion of cells in S-phase as indicated by BrdU staining. Scale bars = 10 μm. Higher magnification of hair cells and supporting cells of the neuromast taken from z-stacks show that hair cells and supporting cells in untreated controls and SP600125-treated animals had no observable morphological differences though there were fewer GFP-positive and Sox2-positive cells in the neuromasts of larvae treated with SP600125. (**E**) Quantification of the number of hair cells in control and SP600125-treated larvae at 24 hours and 48 hours after neomycin incubation. (**F**) Quantification of replicating cells in control and SP600125-treated larvae at 24 hours and 48 hours after neomycin incubation. (**G**) Quantification of the number of Sox2-positive cells in control and SP600125-treated larvae at 24 hours and 48 hours after neomycin incubation. In the 24-hour group, *n* = 100 control neuromasts, *n* = 40 5 μM SP600125-treated neuromasts, *n* = 60 10 μM SP600125-treated neuromasts, and *n* = 32 15 μM SP600125-treated neuromasts. In the 48-hour group, *n* = 72 control neuromasts, *n* = 28 5 μM SP600125-treated neuromasts, *n* = 32 10 μM SP600125-treated neuromasts, and *n* = 32 15 μM SP600125-treated neuromasts. *p < 0.05. (24-hour group: One-way ANOVA; GFP+ cells: F3, 228 = 71.15, *p* < 0.05; Sox2+ cells: F3, 228 = 38.48, *p* < 0.05; BrdU+ cells: F3, 228 = 172.5, *p <* 0.05. 48-hour group: One-way ANOVA; GFP+ cells: F3, 160 = 184.9, *p* < 0.05; Sox2+ cells: F3, 160 = 90.65, *p* < 0.05; BrdU+ cells: F3, 160 = 365.5, *p <* 0.05). Bars are mean ± s.e.m. (**H**, I) Quantification of the ratio of BrdU-positive hair cells and the ratio of BrdU-positive supporting cells in control and SP600125-treated larvae at 24 hours and 48 hours after neomycin incubation. Bars are mean ± s.e.m. In the 24-hour group, *n* = 100 control neuromasts and *n* = 60 10 μM SP600125-treated neuromasts. In the 48-hour group, *n* = 72 control neuromasts and *n* = 32 10 μM SP600125-treated neuromasts. **p < 0.001. (24-hour group: BrdU+ HCs: unpaired *t* test, two-tailed, *t* = 11.54, df = 158, *p <* 0.001; BrdU+ SCs: unpaired *t* test, two-tailed, *t* = 10.5, df = 158, *p <* 0.001. 48-hour group: BrdU+ HCs: unpaired *t* test, two-tailed, *t* = 16.74, df = 102, *p <* 0.001; BrdU+ SCs: unpaired *t* test, two-tailed, *t* = 4.922, df = 102, *p <* 0.001). Bars are mean ± s.e.m.

After 400 μM neomycin treatment for 1 h, most of the hair cells in the lateral line were eliminated, but regeneration occurred rapidly over the following 48 h. To investigate the effect of JNK inhibition on hair cell regeneration, neomycin-treated larvae were placed in 6-well plates and exposed to different doses of SP600125 during recovery periods of 24 h or 48 h. Specific labelling of newly generated hair cells was confirmed using the transgenic zebrafish line *Brn3c*:mGFP. Our results showed that SP600125 significantly decreased the number of regenerated hair cells after neomycin damage. In the 24 h group, 5.66 ± 0.11 GFP-positive hair cells were found in neuromasts (*n* = 100) of the control larvae (Figure 1A2), but the mean value of GFP-positive hair cells per neuromast was 4.8 ± 0.22 (*n* = 40), 3.62 ± 0.15 (*n* = 60), and 2.91 ± 0.15 (*n* = 32) in the 5 μM treated, 10 μM treated (Figure 1B2), and 15 μM treated fish, respectively (Figure 1E; *p <* 0.05). At 48 h post-treatment, there were apparent differences in the number of regenerated hair cells between the untreated larvae and the larvae treated with SP600125. The mean number of GFP-positive hair cells per neuromast was 10.64 ± 0.18 in untreated fish (*n* = 72; Figure 1C2), 7.46 ± 0.25 (*n* = 28) in 5 μM treated fish, 5.81 ± 0.18 (*n* = 32) in 10 μM treated fish (Figure 1D2), and 4.59 ± 0.24 (*n* = 32) in 15 μM treated fish (Figure 1E; *p <* 0.05). Therefore, we conclude that the hair cell regeneration process in larval neuromasts is severely impaired in the presence of SP600125.

### JNK inhibition decreases cellular proliferation in neuromasts

Because the non-sensory supporting cells within the neuromast are the major source of newly regenerated sensory hair cells after neomycin injury [6, 12], we next determined whether SP600125 has any effect on the proliferation of hair cells in neuromasts during the regeneration phase. After neomycin damage, 5 dpf zebrafish larvae were incubated in fresh egg water containing 10 mM BrdU with or without SP600125 at different doses for 24 h or for 48 h. By BrdU incorporation, we observed that the regeneration-associated cell proliferation was significantly inhibited by inhibiting JNK signalling with SP600125. Among the 24 h groups, fewer BrdU-labelled cells were found in SP600125-treated groups compared with the controls (Figure 1A4, 1B4, and 1F; *p <* 0.05). After 48 h of continuous BrdU incorporation, there were significant differences in the number of BrdU-positive cells per neuromast between the control larvae and the larvae treated with SP600125 (Figure 1C4, D4, and 1F; *p <* 0.05) indicating that SP600125 significantly decreased the proportion of neuromast cells undergoing active cell division.

To distinguish the new mitotically regenerated hair cells from cell proliferation, we double-labelled the zebrafish larvae with anti-BrdU and anti-GFP antibodies at 24 h and 48 h after neomycin damage. Our analysis showed that SP600125-treated larvae had fewer BrdU-positive hair cells in the regenerating neuromasts. In control larvae at 24 h post-treatment, the ratio of BrdU and GFP double-labelled cells to the total number of GFP-positive hair cells in the neuromast was 0.46 ± 0.016 (*n* = 100 neuromasts), while the ratio in 10 μM SP600125-treated fish was significantly lower at 0.12 ± 0.026 (*n* = 60 neuromasts, *p <* 0.001) (Figure 1H). Among the 48 h groups, a significant increase in BrdU-positive and GFP-positive hair cells was observed in control fish, while there was a significant decrease in BrdU-positive hair cells per neuromast after exposure to 10 μM SP600125 (Figure 1H; *p <* 0.001).

To further explore the effect of JNK inhibition, we quantitatively assessed the Sox2-labeled supporting cells in the neuromasts after treatment with different concentrations of SP600125 and observed a dose-dependent reduction in the number of stained supporting cells (Figure 1A3, 1B3, 1C3, 1D3 and 1G; *p <* 0.001). We next evaluated the rates of supporting cell division among different groups after neomycin insult by BrdU and Sox2 immunolabelling. After both 24 h and 48 h of regeneration, the ratio of BrdU and Sox2 double-labelled cells to the total number of Sox2-labeled cells in the 10 μM SP600125-treated neuromasts was drastically reduced (Figure 1A3, 1B3, 1C3, 1D3 and 1I; *p <* 0.001), and this most likely explains the reduction in the number of newly regenerated hair cells in the SP600125-treated fish. Taken together, these results show that JNK inhibition has a significant negative impact on proliferation in the regenerating neuromast.

### JNK inhibition induces apoptosis in neuromast cells

**Figure 2:**
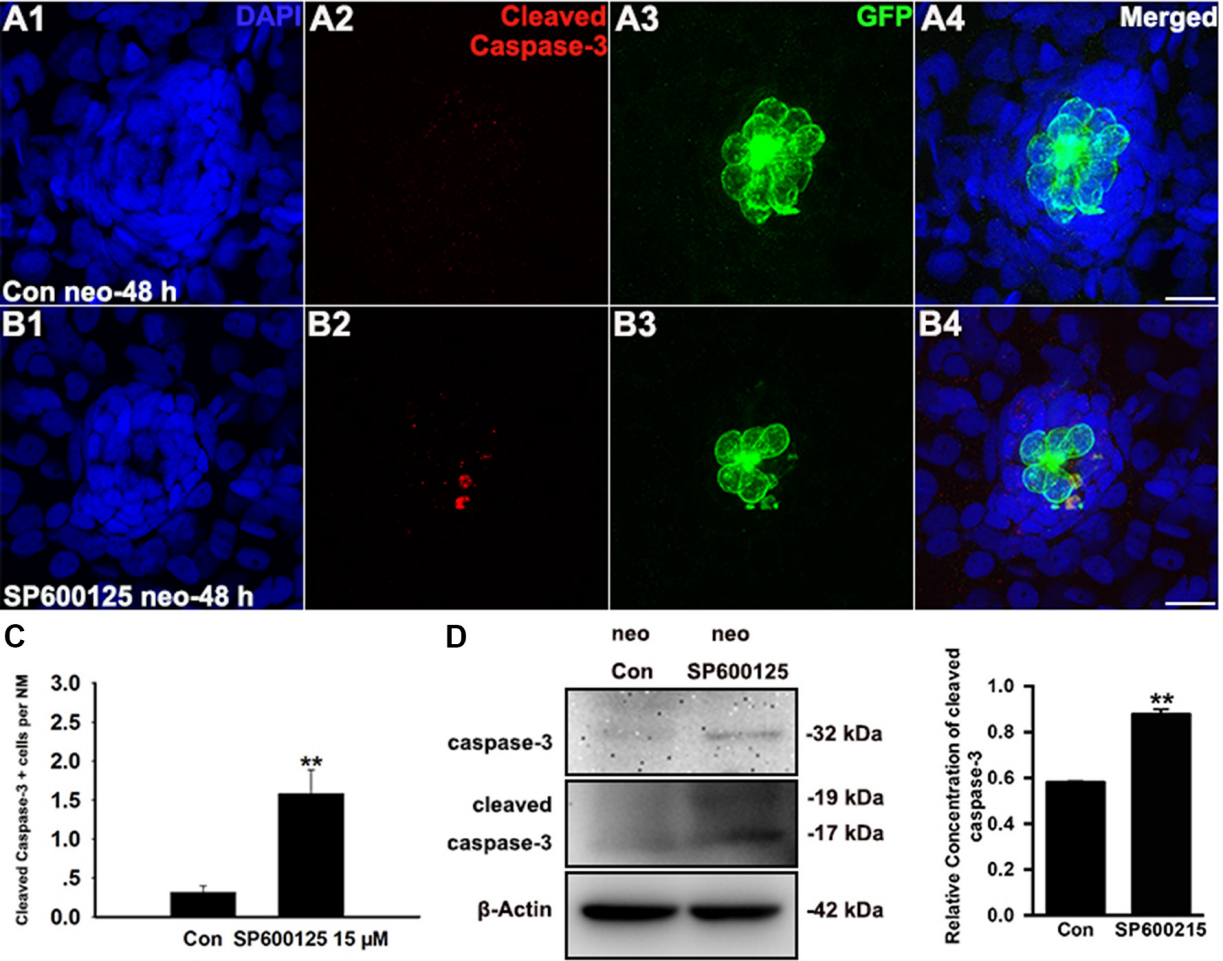
SP600125 induces apoptosis in neuromasts (**A**–**B**) Cleaved caspase-3 staining in the neuromast from a control larva (A) and 15 μM SP600125-treated larvae (B). Scale bar = 10 μm. (**C**) SP600125 treatment increased the numbers of cleaved caspase-3-positive cells. Bars are mean ± s.e.m. *n* = 48 control neuromasts and *n* = 48 15 μM SP600125-treated neuromasts. **p < 0.001. (unpaired *t* test, two-tailed, *t* = 4.051, df = 94, *p <* 0.001). (**D**) After treatment of larvae with 15 μM SP600125 for 48 h, protein extracts were prepared and subjected to western blot assay using antibodies against caspase-3 and cleaved caspase-3. β-Actin was included as the loading control.

In addition to its role in regenerative cell proliferation, JNK signalling has also been implicated in cellular apoptosis [36]. To evaluate the effect of SP600125 on apoptosis, we labelled zebrafish larvae with anti-cleaved caspase-3 antibody. As shown in Figure 2A, we occasionally detected cleaved caspase-3-positive cells in controls. However, the emergence of cleaved caspase-3-positive cells became frequent in larvae treated with 15 μM SP600125 for 48 h (Figure 2B, 2C; *p <* 0.001). This was shown in the western blot analysis of proteins from zebrafish larvae (Figure 2D). However, we cannot rule out the possibility that these proteins might also be affected in other tissues where JNK is expressed because the proteins used for immunoblot analysis are isolated from the whole larvae, not only the neuromasts. We further performed a TUNEL analysis on control and SP600125-treated larvae to investigate the roles of JNK in apoptosis. At 48 h post-treatment, 15 μM SP600125-treated larvae had significantly greater numbers of TUNEL-positive cells when compared to controls (Supplementary Figure S1). Thus, apoptosis appears to contribute to the regeneration defect caused by the JNK inhibitor.

### JNK inhibition inhibits the Wnt signalling pathway

**Figure 3:**
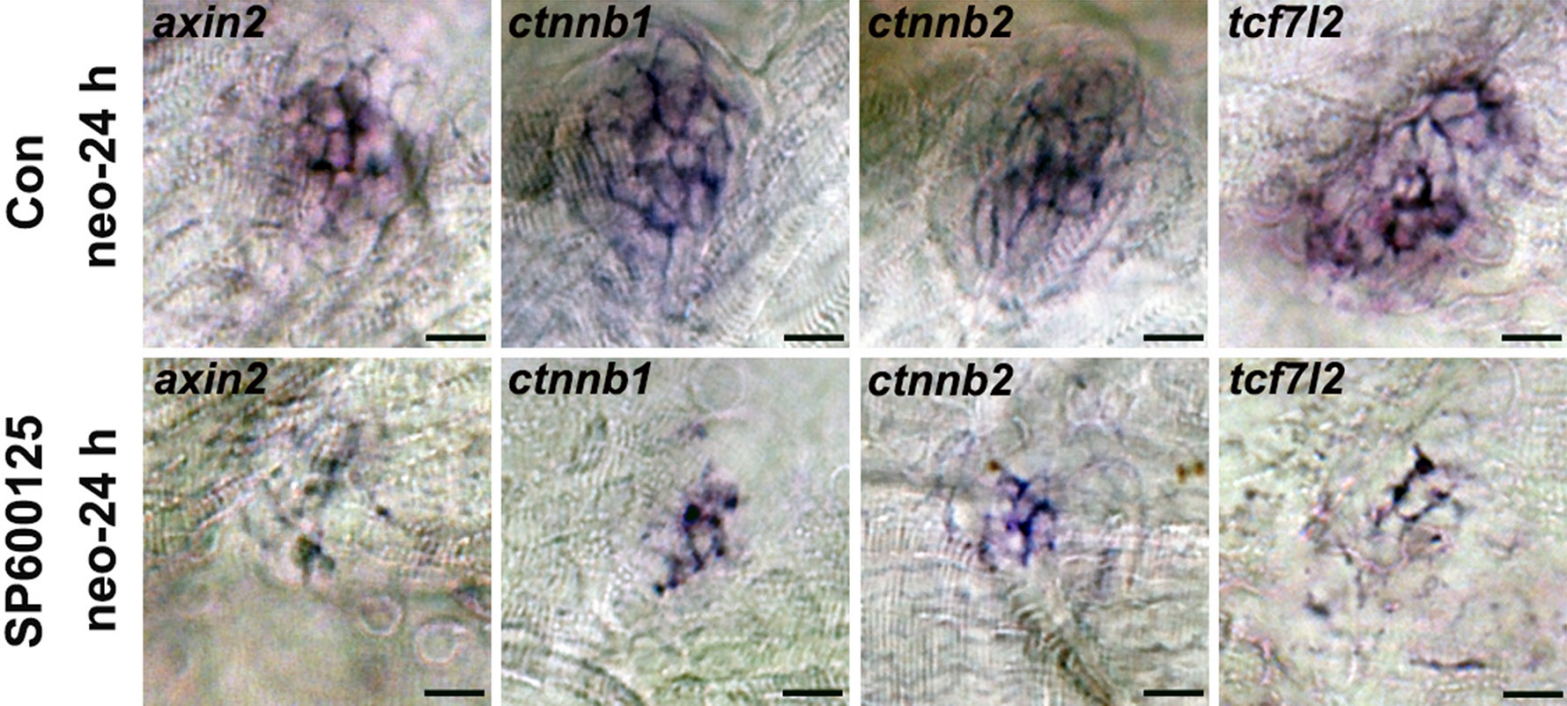
*In situ* hybridization of Wnt pathway-related genes in SP600125-treated and untreated larvae at 24 h after neomycin treatment Expression of *axin2*, *ctnnb1*, *ctnnb2*, and *tcf7l*2 is significantly decreased in SP600125-treated neuromasts during the regeneration period. (*axin2*: *n* = 28 neuromasts from control animals, *n* = 20 neuromasts from SP600125-treated larvae; *ctnnb1*: *n* = 20 neuromasts from control animals, *n* = 16 neuromasts from SP600125-treated larvae; *ctnnb2*: *n* = 18 neuromasts from control animals, *n* = 16 neuromasts from SP600125-treated larvae; *tcf7l2*: *n* = 22 neuromasts from control animals, *n* = 20 neuromasts from SP600125-treated larvae). Results from one representative neuromast are shown. Scale bar = 10 μm.

Wnt signalling is an important player in controlling the regeneration period, and a number of genes have been shown to be required for this process by potentially interacting with this pathway [37–39]. Thus we investigated whether JNK inhibition reduces hair cell regeneration by disrupting the regulation of the Wnt pathway in the neuromast (Figure 3). We examined the expression of the Wnt/β-catenin targets *axin2*, *β-catenin1*, and *β-catenin2* (*ctnnb1* and *ctnnb2* according to the Zebrafish Information Network) as well as β-catenin transcription factor *tcf7l2* in the neuromast using *in situ* hybridization. After neomycin treatment, *axin2*, *ctnnb1*, *ctnnb2*, and *tcf7l2* were up-regulated in the control larvae, indicating that the Wnt pathway is active during hair cell regeneration. In contrast, treatment with SP600125 after exposure to neomycin resulted in a striking down-regulation of the expression of these genes, which was confirmed by the western blot analysis (Supplementary Figure S2). To investigate these findings in more detail, we performed *in situ* hybridization experiments with Wnt/β-catenin pathway genes in non-neomycin-treated control larvae and SP600125-treated larvae. Our analysis showed that larvae with undamaged hair cells showed no significant difference in the expression of Wnt/β-catenin signaling components at the time examined with or without SP600125 treatment (Supplementary Figure S3).

**Figure 4:**
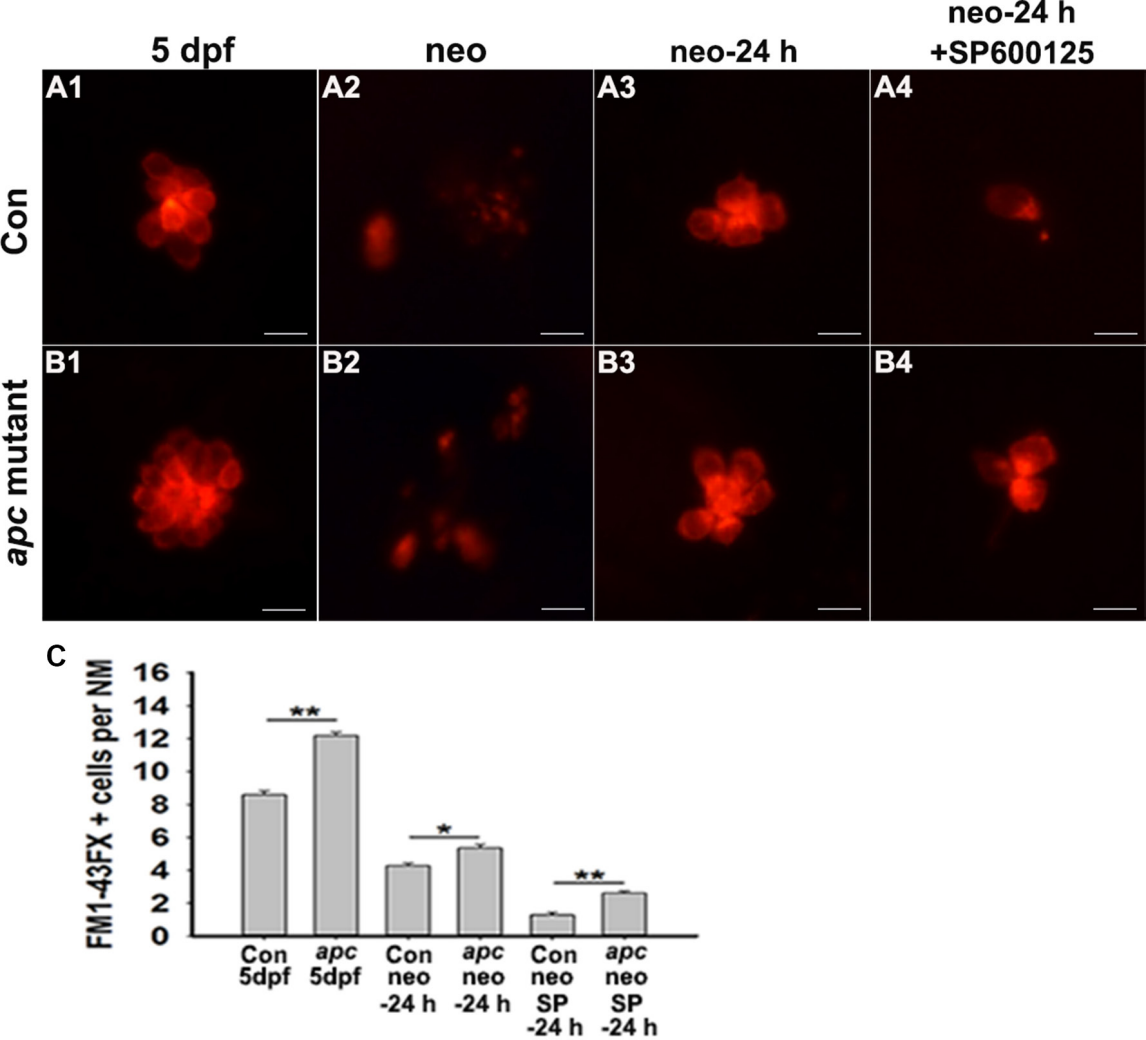
Decreased hair cell regeneration in JNK inhibition zebrafish can be partly rescued by over-activating Wnt signalling (**A**–**B**) The FM1-43FX labelled hair cells are significantly increased in *apc* mutant zebrafish compared to control larvae in SP600125-treated and untreated larvae at 24 h after neomycin treatment. Scale bar = 10 μm. (**C**) Quantification of FM1-43FX-positive hair cells. *p < 0.05; **p < 0.001. (*n* = 53 neuromasts from the non-neomycin-treated 5 dpf control animals, *n* = 44 neuromasts from the non-neomycin-treated *apc* mutant zebrafish at 5 dpf, *n* = 72 neuromasts from the control larvae at 24 h following neomycin damage, *n* = 11 neuromasts from *apc* mutant larvae at 24 h following neomycin damage, *n* = 21 neuromasts from 15 μM SP600125-treated larvae at 24 h following neomycin damage, *n* = 63 neuromasts from 15 μM SP600125-treated *apc* mutant larvae at 24 h following neomycin damage. Con 5 dpf vs. *apc* mutant 5 dpf: unpaired *t* test, two-tailed, *t* = 11.39, df = 95, *p* < 0.001; Con neo-24 h vs. *apc neo*-24 h: unpaired *t* test, two-tailed, *t* = 2.767, df = 81, *p* = 0.007; Con neo SP600125-24 h vs. *apc* neo SP600125-24 h: unpaired *t* test, two-tailed, *t* = 5.186, df = 82, *p* < 0.0001).

These results led to the possibility that JNK supports hair cell regeneration by regulating the Wnt signaling pathway, and we hypothesized that overactive Wnt signalling should rescue the JNK inhibition phenotype. For these studies, we used *apc* mutant zebrafish in which loss of *apc* leads to constitutive Wnt signalling activation. We stained the *apc* mutants with FM1-43FX to determine if hair cell mechanotransduction channels were functional. As shown in Figure 4, at 24 h post-treatment, Wnt overexpression partly reversed the effect of SP600125 on hair cell regeneration. We wanted to determine whether exogenous activation of the Wnt pathway could rescue the effect of SP600125 on hair cell regeneration. To test this, the canonical Wnt activator BIO (1 μM) was applied during the regenerative process following neomycin-induced hair cell death along with either 10 μM SP600125 or DMSO as the control treatments. At 24 h post-treatment, neuromasts in both types of neomycin-treated larvae (neomycin alone or neomycin/SP600125) contained significantly fewer hair cells than neomycin/BIO larvae (Supplementary Figure S4; *p <* 0.0001) indicating that BIO treatment has a significant effect on hair cell regeneration. Furthermore, neomycin/BIO/SP600125-treated larvae had significantly more hair cells in neuromasts when compared with neomycin/SP600125-treated animals (Supplementary Figure S4; *p <* 0.05), but had fewer hair cells when compared with neomycin-treated animals (Supplementary Figure S4; *p <* 0.0001), indicating that the SP600125-induced hair cell regeneration defect could be partly rescued by BIO treatment. These results are consistent with rescue studies using *apc* mutants. All these findings suggested that JNK might support hair cell regeneration by controlling the Wnt pathway.

## DISCUSSION

Previous work in zebrafish showed that JNK activity is required for regeneration-associated cell proliferation and cell cycle activation of the blastema cells [40]. However, little was known regarding the effect of JNK inhibition on hair cell regeneration. In this report, we provide evidence that JNK supports hair cell regeneration in larval neuromasts and that JNK inhibition by SP600125 results in fewer hair cells and supporting cells in regenerative neuromasts.

Several studies have suggested a role for the JNK pathway in cell-cycle regulation [19, 21], and the JNK inhibitor SP600125 is well known to induce G_2_/M phase cell-cycle arrest and apoptosis in various cancer cells [24, 25, 41]. Our results agree well with previous reports showing that inhibition of JNK by SP600125 leads to a drastic decrease of proliferation as assessed by BrdU staining in zebrafish. Given that the cell proliferation defect in the regenerating neuromasts of SP600125-treated fish could also be due to induction of apoptosis, we performed a cell death analysis by TUNEL analysis and cleaved caspase-3 staining. Our present data clearly show that SP600125 treatment significantly induced apoptosis in the neuromasts.

Recent studies have suggested that the Wnt signalling pathway plays pivotal roles in a wide variety of developmental processes by controlling cell proliferation, cell fate determination, and several cellular polarization events [42–45], and it is also heavily implicated in regeneration processes in several tissues [46, 47]. In this report, it has been shown that Wnt pathway genes *axin2*, *ctnnb1*, *ctnnb2*, and *tcf7l2* are stimulated in neuromast cells at 24 h of regeneration, and this supports previous studies that Wnt signalling activation increases the degree of hair cell regeneration after damage [46, 47, 50]. However, JNK inhibition with SP600125 decreases the expression of these Wnt signalling genes, raising the possibility that activated Wnt signalling might cooperate with the JNK pathway to support hair cell regeneration. Our results suggest that inducing constitutively active Wnt either in *apc* mutant zebrafish or with BIO treatment could partly rescue the hair cell-less phenotype caused by abrogation of JNK signalling during regeneration. However, we did not observe complete regeneration in neomycin/SP600125-treated *apc* mutants or in neomycin/SP600125-treated/BIO-recovery larvae, which suggests that other molecular mechanisms or signaling pathways might be involved in the JNK-induced hair cell regeneration.

Many extensive cross-regulatory interactions between JNK and both canonical and noncanonical Wnt signalling pathways have been reported previously in a number of processes in many organisms, including both the promotion and repression of Wnt activity [51–55] For example, previous work has shown that Wnt5a, a noncanonical Wnt ligand, is capable of activating JNK. Appropriate activation of JNK is able to function downstream of Wnt5a to correct convergent extension movements in *Xenopus* [56]. Wnt7a stimulates JNK activation and c-Jun phosphorylation in non-small cell lung cancer cells [57]. More evidence for the well-characterized crosstalk between JNK and the canonical Wnt signalling pathway has now appeared in mammalian cells and zebrafish [51, 58]. The connection between these two pathways might result in multiple correlations of expression among phosphorylated c-Jun (p-c-Jun), TCF4, and β-catenin target genes. Several groups have reported that the p-c-Jun protein cooperates with the HMG family transcription factor TCF4 to form a functional transcription complex that cooperatively enhances their transcriptional activity in the presence of β-catenin, and this complex plays a critical role in mouse intestinal tumor development. Abrogation of JNK signalling by pharmacological inhibition leads to a failure in c-Jun phosphorylation and inhibition of the phosphorylation-dependent interactions between c-Jun and TCF4 [58]. In addition, Gan and colleagues demonstrated a novel role of JNK signalling in regulating the Wnt signalling via direct interaction of c-Jun with Dishevelled, a pivotal regulator of the canonical Wnt signalling pathway. c-Jun can function as a scaffold protein within the β-catenin–TCF transcription complex to bridge Dvl to TCF on the Wnt target gene promoters and stimulate gene transcription [51]. The crosstalk between JNK and the canonical Wnt pathways in regeneration processes will provide important directions for future studies regarding how JNK regulates hair cell regeneration.

In conclusion, our present study supports a novel role for JNK in hair cell regeneration. JNK inhibition with SP600125 decreases hair cell regeneration in larval zebrafish, an this defect can be partly rescued by over-activation of the Wnt signalling pathway. Taken together, our study provides new insights into the mechanisms of hair cell regeneration in the zebrafish lateral line and proposes that JNK activation might represent a useful therapeutic strategy in the treatment of hearing loss.

## MATERIALS AND METHODS

### Zebrafish embryos and drug administration

Zebrafish embryos were maintained in our facility according to standard procedures. The *apc* mutant transgenic line was obtained from Professor Xu Wang. The ages of the zebrafish larvae are described as days post fertilization (dpf). SP600125 (Sigma-Aldrich, St Louis, MO, USA) was dissolved in dimethyl sulfoxide (DMSO, Sigma-Aldrich) at a stock concentration of 50 mM and further diluted to the desired concentrations in fresh egg water. Dose-response data were obtained by treating larvae with SP600125 (5 μM, 10 μM, and 15 μM) after hair cell damage. For hair cell damage, neomycin sulphate (Sigma-Aldrich) was added to 5 dpf larvae at a final concentration of 400 μM and incubated for 1 h. This was followed by three rinses in fresh egg water, and the zebrafish larvae were allowed to recover for 24 h or 48 h at 28.5°C.

### Cell proliferation and analysis

Proliferating cells in the lateral line neuromasts were labelled by adding 10 mM 5-bromo-2-deoxyuridine (BrdU; Sigma-Aldrich) to the fresh egg water for 24 h or 48 h at 28.5°C. Larvae were then fixed with 4% PFA overnight at 4°C, and BrdU incorporation was detected by fluorescent immunostaining. The fixed larvae were washed three times in PBS containing 0.5% Triton X-100 (PBT-2) and placed in 2 N HCl for 0.5 h at 37°C. Larvae were blocked in 10% normal goat serum for 1 h at room temperature and incubated with the monoclonal primary anti-BrdU antibody (1:200 dilution; Santa Cruz, Dallas, TX, USA. Cat. no. sc-32323) overnight at 4°C. The next day, larvae were washed three times for 10 min each with PBT-2 and then incubated with the secondary antibody for 1 h at 37°C. Fluorescently labelled larvae were imaged with a Leica confocal fluorescence microscope (TCS SP5; Leica, Wetzlar, Germany). Images were processed using Photoshop software (Adobe).

### Immunohistochemistry

For immunohistochemistry analysis, larvae were fixed with 4% PFA and were permeabilized with PBT-2 for 30 min followed by incubation in blocking solution for 1 h. The following antibodies were used as primary antibodies: anti-GFP (1:1000 dilution; Abcam, Cambridge, UK); anti-Sox2 (1:200 dilution; Abcam); and anti-cleaved caspase-3 (1:500 dilution; Cell Signaling Technology Inc., Danvers, MA, USA). The larvae were washed three times with PBT-2 and incubated with secondary antibodies to detect primary antibodies. Nuclei were labelled with 4,6-diamidino-2- phenylindole (DAPI; 1:800 dilution; Invitrogen, Carlsbad, CA, USA) for 20 min at room temperature.

### FM1-43FX labelling

To visualize and image the hair cells in lateral line neuromasts, the vital dye FM1-43FX (Molecular Probes, Eugene, OR, USA) was applied at a concentration of 3 μM to live larvae for 45 s in the dark. After quickly rinsing three times with fresh water, the larvae were anesthetized in 0.02% MS-222 and fixed with 4% PFA in PBS for 2 h at room temperature or overnight at 4°C.

### Western blot analysis

Total protein was isolated with the AllPrep DNA/RNA/Protein Mini Kit (QIAGEN, Hilden, Germany) according to the manufacturer's instructions. Protein concentrations were measured using a BCA protein kit (Thermo Fisher Scientific, Rockford, IL), and proteins were separated on SDS-polyacrylamide gels and transferred onto PVDF membranes (Immobilon-P; Millipore, Bedford, MA, USA). The membranes were blocked with 5% nonfat dried milk in TBST (50 mM Tris-HCl (pH 7.4), 150 mM NaCl, and 0.1% Tween-20) for 1 h at room temperature and then blotted overnight with primary antibodies at 4°C. The following antibodies were used as primary antibodies: anti-cleaved caspase-3 (1:1000 dilution; Cell Signaling Technology), anti-caspase-3 (1:1000 dilution; Abcam), anti-axin2 (1:1000 dilution; Abcam), and anti-tcf7l2 (1:1000 dilution; Abcam).

### Whole-mount *in situ* hybridization

The probes used in *in situ* hybridization (*axin2*, *ctnnb1*, *ctnnb2*, and *tcf7l2*) were amplified by PCR from zebrafish embryo cDNA using the following primers and cloned into the pGEM-T Easy Vector (Promega, cat. no. A1360): *axin2* forward: 5′-accgacaaaccaagcaca ag-3′; *axin2* reverse: 5′-tccgttttgagttatgaagctct-3′; *ctnnb1* forward: 5′-cccaggactacaagaagcga-3′; *ctnnb1* reverse: 5′-acaggcaaggctaaggttga-3′; *ctnnb2* forward: 5′-catcgag aacatccagcgtg-3′;*ctnnb2* reverse: 5′-tggactacactacagcc gtc-3′; *tcf7l2* forward: 5′-ccctccacatctacagggag-3′; *tcf7l2* reverse: 5′-tgtgttcattgccctctcct-3′. Digoxigenin-labeled antisense RNA probes were generated by *in vitro* transcription using T7 or SP6 RNA polymerase. Regular whole-mount *in situ* hybridization of zebrafish embryos was performed as previously described [17, 59]. Details of the probes used are available on request. Briefly, the embryos were depigmented with 1-phenyl-2-thiourea (PTU, Sigma-Aldrich, cat. no. P7629), euthanized in MS-222, and fixed overnight with 4% PFA at 4°C. The fixed embryos were washed in PBS with 0.1% Tween-20 (PBST) and placed in 100% methanol at −20°C for dehydration. Prior to use, they were rehydrated in a graded methanol series and washed three times for 5 min with PBST. To permeabilize the embryos, proteinase K (10 μg/mL in PBST) was added for 50 min and the embryos were refixed in 4% PFA for 20 min. After washing in PBST, the embryos were prehybridized at 65°C for ≥ 2 h in hybridization buffer. For hybridization, the labeled probes were added to the hybridization buffer at 65°C overnight. After washing for 15 min with 75%, 50%, and 25% hybridization buffer and 2× SSCT (20× SSC, Life technologies, AM9770; 0.1% Tween-20) and for 30 min twice in 0.2× SSC at 65°C, embryos were blocked for at least 2 h at 4°C in blocking buffer (Roche cat. no._11096176001) and were incubated with preabsorbed sheep anti-digoxigenin-AP Fab fragments (Roche cat. no. 11093274910) at a 1:4000 dilution in blocking buffer overnight at 4°C. The next day, the embryos were washed 4 × 30 min with 2 mg/mL BSA in PBST and 3 × 5 min in staining buffer (100 mM Tris (pH 9.5), 100 mM NaCl, and 0.1% Tween-20). Afterwards, the embryos were stained with BM purple AP substrate (Roche cat. no. 11 442074001) in the dark. Finally, the color reaction was stopped by adding PBST, and the embryos were observed under a bright field microscope (Nikon Instruments).

### TUNEL staining

For TUNEL (Terminal deoxynucleotidyl transferase-mediated dUTP nick end labeling) assays, larvae were incubated in 0.1 M glycine/PBS solution for 10 min and then rinsed with PBT-2 three times for 10 minutes each. The larvae were then processed using the *In Situ* Cell Death Detection Kit (Roche, Nutlet, NJ, USA; cat. no.11684795910) following the directions supplied by the manufacturer.

### Statistical analysis

Prior to analysis, all data were first examined for normality and homogeneity of variances by the Shapiro–Wilk test and Levene's test, respectively. For statistical comparisons, differences among groups were compared using one-way ANOVA, and differences between groups were compared using an unpaired *t*-test (2-tail) (see figure legends for details). Data were analyzed using SigmaPlot (version 12.0 for Windows; Systat Software Inc., CA, USA). All data are presented as the mean ± s.e.m. A *p*-value < 0.05 was considered statistically significant, and *p <* 0.001 was considered highly significant.

## SUPPLEMENTARY MATERIALS AND FIGURES


