# Supplementary material for: Effect of JNK inhibitor SP600125 on hair cell regeneration in zebrafish (Danio rerio) larvae
Source: Oncotarget. 2016 Jul 12;7(32):51640–50. doi: 10.18632/oncotarget.10540 (PMC5239503; doi:10.18632/oncotarget.10540)
Supplement: Supplementary file 1 [file oncotarget-07-51640-s001.pdf]

# Effect of JNK inhibitor SP600125 on hair cell regeneration in zebrafish (*Danio rerio*) larvae

## Supplementary Materials

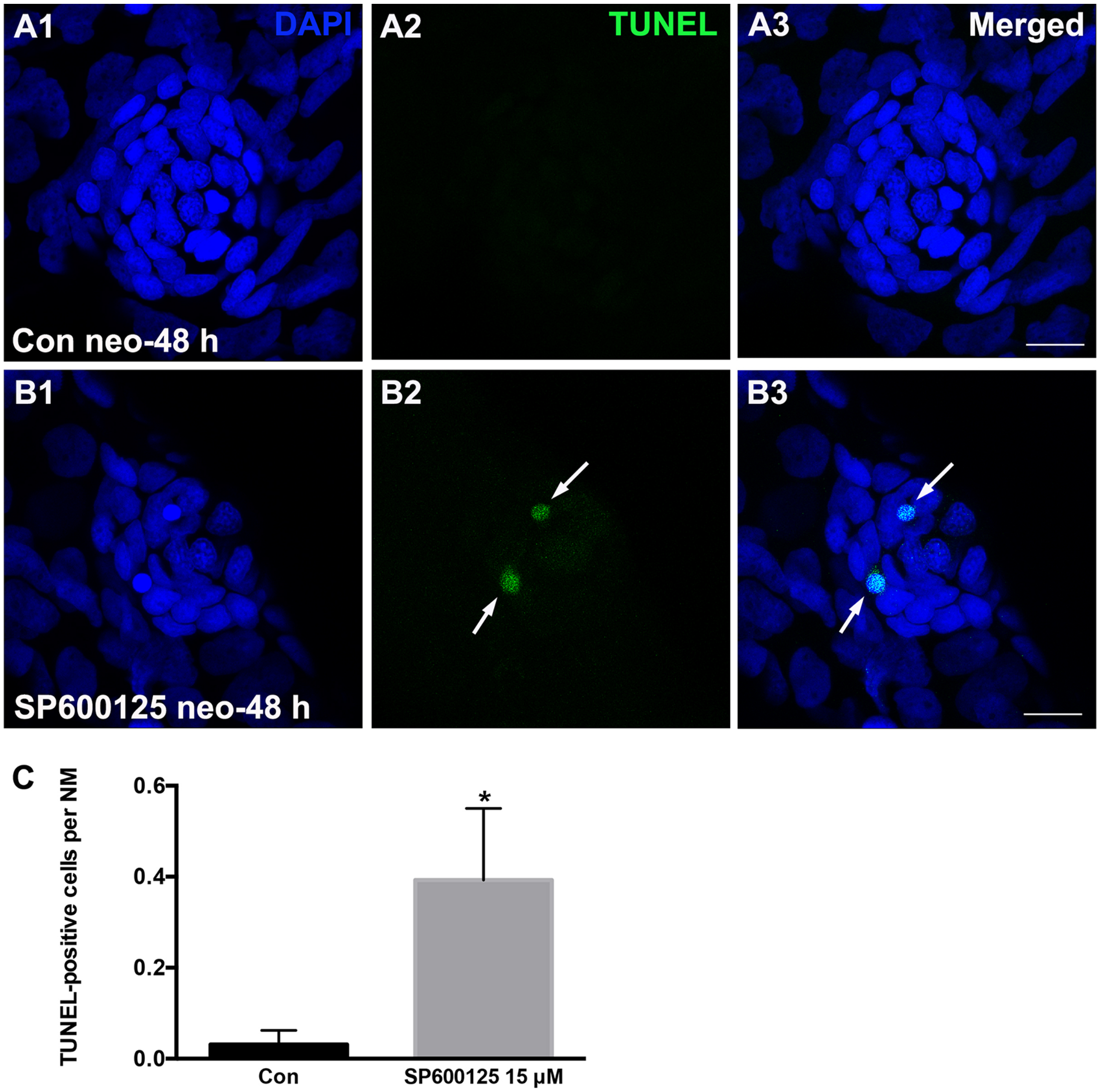


**Supplementary Figure S1: SP600125 induces apoptosis in neuromasts.** (**A–B**) TUNEL staining in the neuromasts of control and SP600125 (15 µM)-treated larva at 48 h following neomycin-induced hair cell death. White arrows indicate TUNEL-positive cells. Scale bar = 10 µm. (**C**) Quantification of apoptosis induced by SP600125 treatment. Data are expressed as mean ± s.e.m, $*p < 0.05$. ($n = 32$ neuromasts from control animals, $n = 28$ neuromasts from 15 µM SP600125-treated animals; unpaired $t$ test, two-tailed, $t = 2.401$, df = 58, $p = 0.0196$).

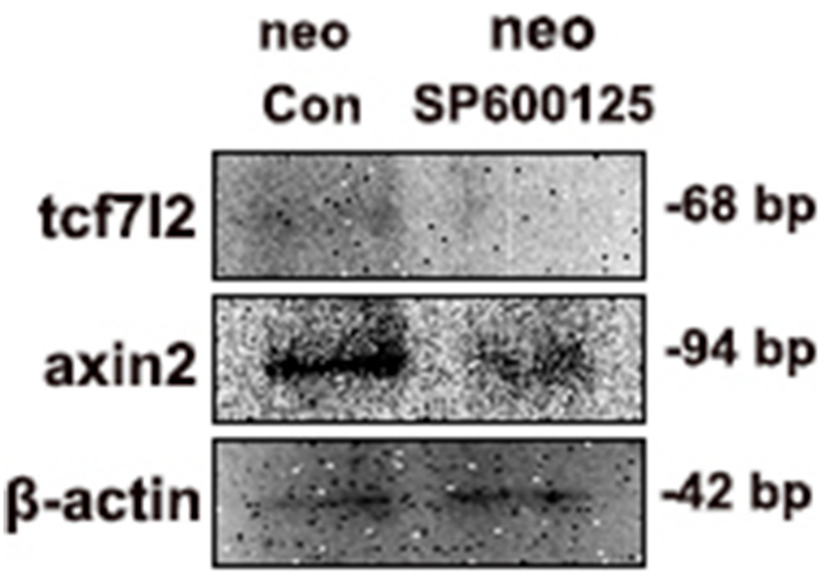


**Supplementary Figure S2: SP600125 inhibits expression of Wnt signaling components.** After treatment of larvae with 15 μM SP600125 for 24 h following neomycin damage, whole protein extracts were immunoblotted using axin2 antibody and tcf7l2 antibody. β-Actin was used as a loading control.

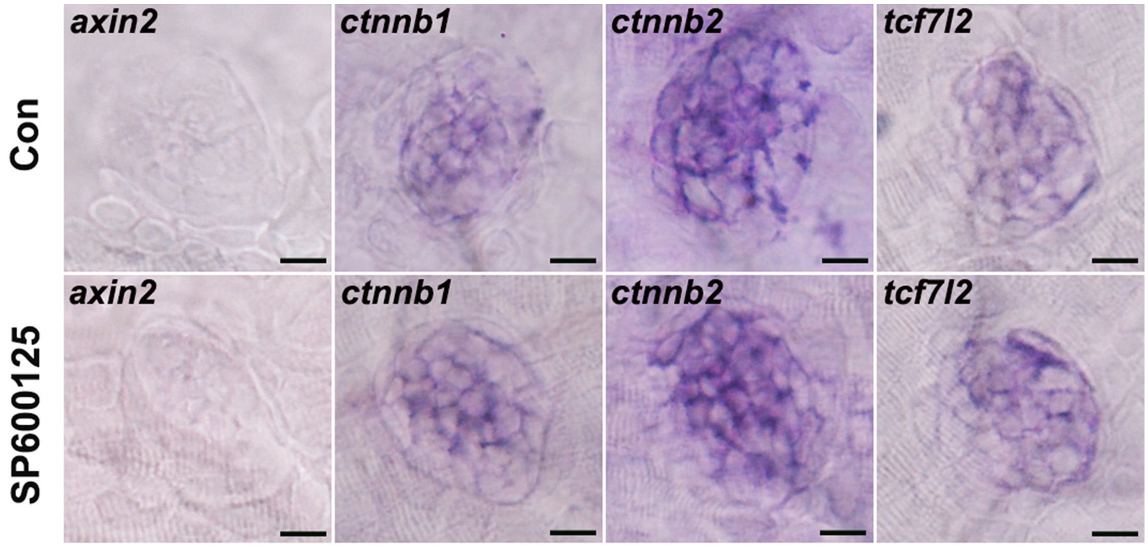


**Supplementary Figure S3: Effect of SP600125 on Wnt pathway-related gene expression in neuromasts.** Expression of *axin2*, *ctnnb1*, *ctnnb2*, and *tcf7l2* in SP600125-treated (24 h) and untreated control larvae without neomycin treatment. (*axin2*: $n = 26$ neuromasts from control animals, $n = 30$ neuromasts from SP600125-treated larvae; *ctnnb1*: $n = 36$ neuromasts from control animals, $n = 28$ neuromasts from SP600125-treated larvae; *ctnnb2*: $n = 32$ neuromasts from control animals, $n = 34$ neuromasts from SP600125-treated larvae; *tcf7l2*: $n = 24$ neuromasts from control animals, $n = 24$ neuromasts from SP600125-treated larvae). Results from one representative neuromast are shown. Scale bar = 10 μm.

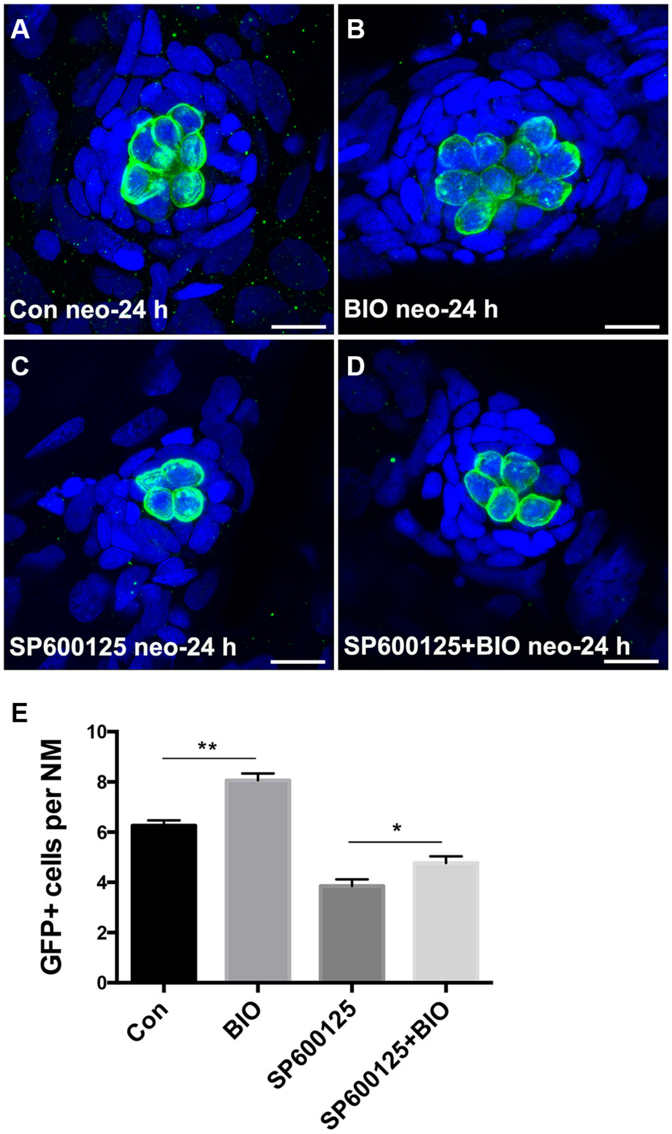


**Supplementary Figure S4: Decreased hair cell regeneration in JNK inhibition zebrafish can be partly rescued by BIO treatment.** (**A**–**D**) BIO/SP600125-treated larvae had significantly more GFP-positive hair cells in neuromasts when compared with SP600125-treated animals at 24 h after neomycin treatment. Scale bar = 10 μm. (**E**) Quantification of GFP-positive hair cells. *$p < 0.05$; **$p < 0.001$. ($n = 38$ neuromasts from control animals, $n = 31$ 1 μM BIO-treated neuromasts, $n = 34$ 10 μM SP600125-treated neuromasts, $n = 31$ SP600125/BIO-treated neuromasts. Con vs. SP600125: unpaired $t$ test, two-tailed, $t = 7.229$, df = 70, $p < 0.0001$; Con vs. BIO: unpaired t test, two-tailed, $t = 5.359$, df = 67, $p < 0.0001$; SP600125 vs. BIO: unpaired t test, two-tailed, $t = 10.99$, df = 63, $p < 0.0001$; SP600125 vs. BIO+SP600125: unpaired $t$ test, two-tailed, $t = 2.456$, df = 63, $p = 0.0168$; BIO+SP600125 vs. Con: unpaired $t$ test, two-tailed, $t = 4.545$, df = 67, $p < 0.0001$).
